# The mitochondrial genome of the Maltese honey bee, *Apis mellifera ruttneri* (Insecta: Hymenoptera: Apidae)

**DOI:** 10.1080/23802359.2020.1717384

**Published:** 2020-01-27

**Authors:** Leigh Boardman, Amin Eimanifar, Rebecca T. Kimball, Edward L. Braun, Stefan Fuchs, Bernd Grünewald, James D. Ellis

**Affiliations:** a Honey Bee Research and Extension Laboratory, Entomology and Nematology Department, University of Florida, Gainesville, FL, USA;; b Department of Biology, University of Florida, Gainesville, FL, USA;; c Institut für Bienenkunde, Goethe-Universität Frankfurt am Main, Oberursel, Germany

**Keywords:** Mitogenome, A-lineage honey bee, next-generation sequencing

## Abstract

The mitochondrial genome of *Apis mellifera ruttneri* consisted of 13 protein-coding genes, two rRNAs, 22 tRNAs, an AT-rich control region, and was 16,577 bp long. The phylogenetic analyses suggested that *A. m. ruttneri* was closely related to two North African subspecies: *A. m. sahariensis* and *A. m. intermissa*.

The Maltese honey bee, *Apis mellifera ruttneri* (Sheppard et al.), is endemic to Malta. Despite this European location, analyses, including using mitochondrial DNA with *Eco*R1 restriction enzyme digestion, suggest that *A. m. ruttneri* forms part of the African (A) lineage - similar to *A. m. sicula* (Sheppard et al. 1997). Here, we present the mitochondrial genome of a worker *A. m. ruttneri* honey bee from San Julian’s, Malta (35°53 N, 14°26 E, collector: Dr. W.S. Sheppard, 1995, Voucher 2050 from Ruttner Bee Collection at the Bee Research Institute at Oberursel, Germany, GenBank: MN714162). Identity of the subspecies was confirmed morphometrically.

Following Eimanifar et al. (2017), genomic DNA was extracted, quantified, and PE-150 bp sequenced on Illumina Hi-Seq 3000/4000 (San Diego, CA). The sequencing data were checked for quality using FastQC (Andrews 2010), and trimmed with Trimmomatic (Bolger et al. 2014). The paired R1 trimmomatic output was mapped to the mitogenome of *A. m. sahariensis* (MF351881) in Geneious Prime 2019.0.4 (Kearse et al. 2012) using the method from Boardman et al. (2020). The assembled mitogenome was annotated in mitos2 (Bernt et al. 2013). The annotated mitogenome was manually adjusted to the *A. m. capensis* annotation (KX870183) in Geneious Prime. Protein-coding genes (PCGs) and ribosomal RNA (rRNA) sequences were manually aligned to sequences from other *Apis* species and *Apis mellifera* subspecies in Mesquite v3.5 (Maddison and Maddison 2018). Phylogenetic comparisons used RAxML 8.2.10 (Stamatakis 2014) with the GTRGAMMA model and 1000 bootstrap replicates (-f a option) on the CIPRES Science Gateway V.3.3 (Miller et al. 2010), and *p*-distances were generated with PAUP 4.0a (Swofford 2003).

The mitochondrial genome of *A. m. ruttneri* was 16,577 bp (43.2% A 9.6% C, 5.6% G, 41.5% T). Among the 13 protein-coding genes, nine were on the light strand (*nad2*, *co1*, *co2*, *atp8*, *atp6*, *co3*, *nad3*, *nad6* and *cytb*) and four were on the heavy strand (*nad1*, *nad4*, *nad4l*, *nad5*). The two *atp* genes, *atp8* and *atp6*, shared 19 nucleotides. The 13 PCGs used a variety of start codons: ATT (*n* = 6), ATG (*n* = 4), ATA (*n* = 2), and ATC (*n* = 1), while all PCGs ended with TAA. The 16S ribosomal RNA (rRNA) was 1,325 bp, with 84.1% AT, while the 12S rRNA was 785 bp with 81.2% AT. There were 22 transfer RNAs located in various positions on the mitogenome. They varied in size from threonine being the longest (80 bp) to serine and glutamine being the shortest (63 bp).

Phylogenetically, *A. m. ruttneri* was closest to other North African honey bees (Figure 1). The subspecies with the lowest *p*-distances from *A. m. ruttneri* were *A. m. sahariensis* (*p* = 0.00114), *A. m. intermissa* (*p* = 0.00129), and *A. m. iberiensis* (*p* = 0.00221). This confirms previous studies suggesting that *A. m. ruttneri* is part of the A-lineage. Given their close geographic proximity, comparable size and color, and similar behavioral characteristics (Sheppard et al. 1997), future research comparing this mitogenome to that of the Sicilian honey bee *A. m. sicula* would be interesting.

**Figure 1.**
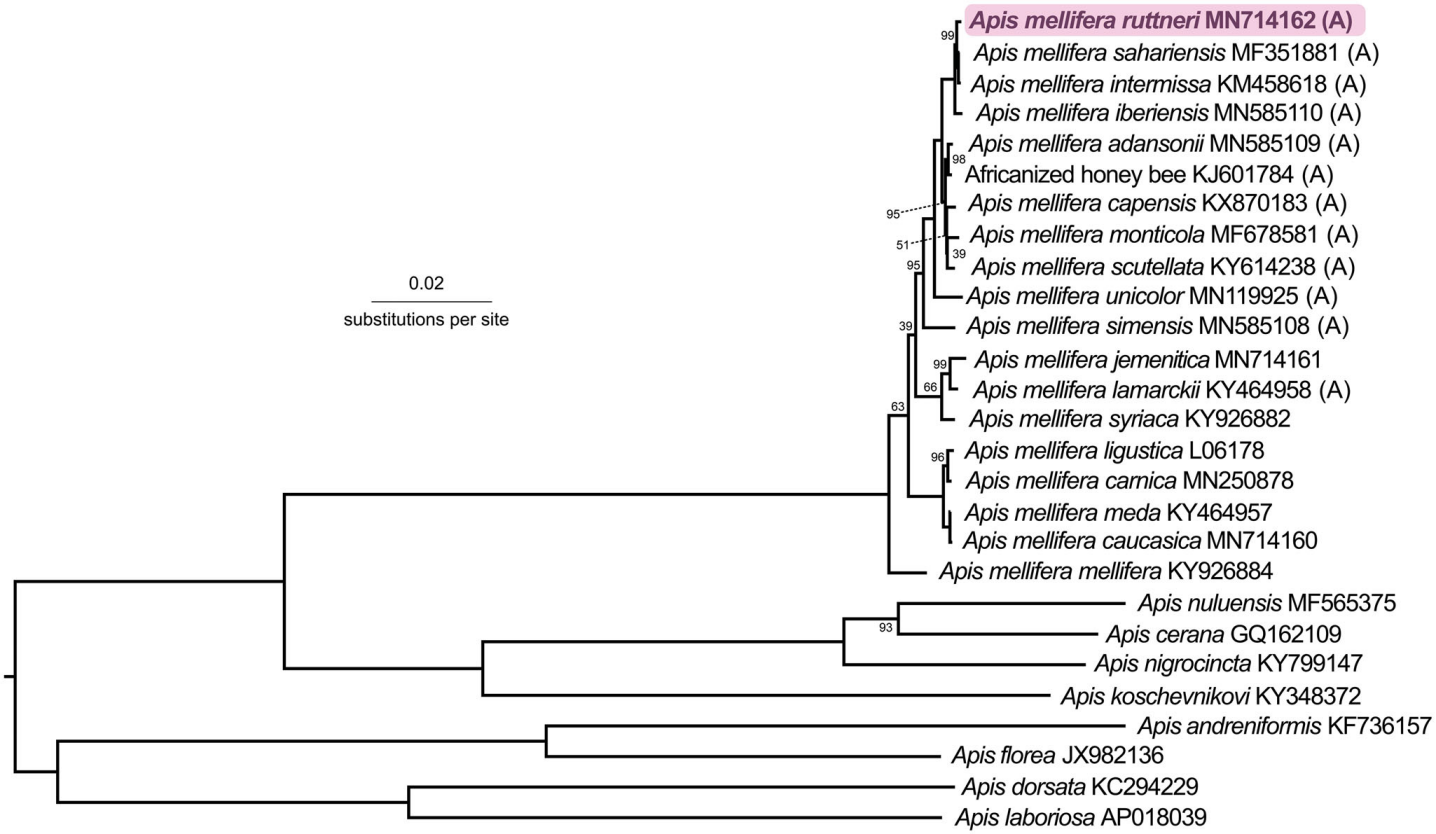
Phylogenetic tree showing the relationship between *Apis mellifera ruttneri* (GenBank: MN714162) and 26 other *Apis* honey bees (GenBank accession numbers are listed after species names). Node labels indicate bootstrap values. Unlabeled lineages are 100%. The (A) indicates subspecies from the African A-lineage.
